# The immune microenviroment in somatotropinomas: from biology to personalized and target therapy

**DOI:** 10.1007/s11154-022-09782-1

**Published:** 2023-01-20

**Authors:** Sabrina Chiloiro, Laura De Marinis

**Affiliations:** 1 UOC Endocrinology and Diabetology, Fondazione Policlinico Universitario A. Gemelli IRCCS, 00168 Roma, Italy; 2 Department of Translational Medicine and Surgery, Università Cattolica del Sacro Cuore, 00168 Roma, Italy

**Keywords:** Acromegaly, Pituitary, Immunotherapy, Target therapy

## Abstract

Pituitary tumors are rare neoplasms, with a heterogeneous biological and clinical behavior, due to their clinical course, local invasive growth, resistance to conventional therapies and the risk of disease progression. Recent studies on tumor microenvironment (TME) provided new knowledge on the biology of these neoplasia, that may explain the different phenotypes of these tumors and suggest new biomarkers able to predict the prognosis and the treatment outcome. The identification of molecular markers that act as targets for biological therapies may open new perspectives in the medical treatments of aggressive pituitary tumors.

In this paper, we will review data of TME and target therapies in somatotropinomas.

## Introduction

Pituitary tumors originating from the endocrine cells of the anterior pituitary account for about 15% of all intra­cranial neoplasms, with an estimated prevalence of around 80-100 cases per 100,000 inhabitants [1].

Despite pituitary tumors being usually considered benign neoplasia, they represent a heterogeneous group of tumors. Their biology ranges from a benign adenoma with an unchanged life expectancy to highly malignant tumors with a limited life expectancy [2].

In order to better underline the heterogeneity of pituitary adenomas, the 5th edition of the WHO Classification of Endocrine and Neuroendocrine Tumor (2022) suggested that the nomenclature of pituitary neuroendocrine tumor (PitNET) be integrated into the historical definition of pituitary adenoma [3].

The prognosis of these tumors is strongly related to their biology. Even in the absence of overt metastases, some pituitary tumors display an almost equally aggressive behavior and are responsible for increased morbidity and mortality, in particular in patients with long term persistence of hormone hypersecretion, such as acromegaly and Cushing disease [2, 4, 5]. In patients affected by secreting pituitary tumors, the prompt control of the tumor mass and hormone hypersecretion

is of central importance for reducing the occurrence of systemic complications and the therefore related mortality [6].

In real-life clinical practice, pituitary adenomas/PitNets are considered aggressive in cases with high hormonal serum levels, invasion of the neighboring anatomical structures (such as the cavernous sinus, the dura mater, the ventricular system, the clivus and other bones), high proliferative activity, rapid growth, poor response to conventional therapies and/or regrowth irrespective of the treatments. Standardized criteria are actually not available for defining aggressive pituitary pituitary adenomas/PitNets [1].

The ESE guidelines define aggressive the invasive tumors with an unusually rapid tumor growth rate or with clinically a relevant tumor growth despite optimal standard therapies, such as surgery, radiotherapy and conventional medical treatments [1]. The 2017 WHO classification also included the radiological invasion as a criteria for the identification of the so called “high risk pituitary adenomas” [7].

The characterization of an aggressive pituitary adenoma/PitNets also includes an histopathological evaluation, that comprises the immunohistochemistry for pituitary transcription factors and hormones, the evaluation of the Ki67 index, the mitotic count and the immune-detection of p53 [8]. Clinical and pathological markers able to predict the tumor behavior are still not clearly defined. The identification of additional prognostic and therapeutic markers will enable personalized and timely therapeutic decisions in the future [9–11], greatly assisting the clinical management of patients. Indeed, if standard therapies fail, temozolomide is recognized as the first line chemotherapy for aggressive pituitary tumors. Other emerging options are mainly molecular target therapies, peptide receptor radionuclide therapy (PRRT) and immunotherapy, which, although promising, have thus far showed limited effectiveness [12, 13].

The tumor microenvironment (TME) is a special milieu generated by the interaction between the tumor cells and the host during the tumor development. The TME affects tumor proliferation, invasiveness and angiogenesis [14]. TME is composed mainly from non-tumor cells, blood vessels, extracellular matrix and soluble factors, such as cytokines and enzymes [15] .

The TME may contribute to explain the heterogeneous behavior of pituitary adenomas/PitNets, through the interplay between tumor cells and TME components. The interaction among pituitary tumor cells and TME components generates a complex networks. The main actions of TME in pituitary adenomas/PitNets are summarized in Table 1.

Somatotropinomas represent an interesting model to investigate the TME: *in vitro* studies proved that the growth hormone (GH), the insulin-like growth factor-I (IGF-I), the prolactin (PRL) and the somatostatin are able to modulate the immune response. Literature data suggest that the IGF-I and GH exert a strong pro-inflammatory effect [16]. A recent study reported that in acromegaly patients the co-stimulation of GH and IGF-I promotes the production of interleukin (IL)-6, alpha tumor necrosis factor (TNF-alpha) and interferon (IFN) from peripheral blood mononuclear cells [17]. At the same time, the somatostatin regulates the proliferation and activity of inflammatory cells, the growth of tumor cells and the plasticity of normal tissue [18].

**Table 1:** TME components in pituitary adenomas/PitNets and their actions.

**TME components**		**Anti-tumor immune response**		**Immune response escape**
**Immune cells**	Tumor-infiltrating lymphocytes (TILs)	Cytotoxic CD8+CD45RO+ memory T cells CD4+ T helper 1 T cells, Innate cytotoxic lymphocytes Natural killer cells	promote a cytotoxic cascade; promote humoral response by an antibody-dependent target cell killing promote the secretion of TNF- α, IFN-γ, IL-2	
		CD4+CD25+Foxp3+ regulatory CD4+ T helper 2 T helper 17 cell		inhibit the effector lymphocytes in both cytokine or both cell-contact dependent fashion promote the secretion of IL-4, IL-5, IL-6, IL-10, and IL-13
	Tumor-associated macrophages (TAMs)	M1-TAMs (CD80, CD86, MHC II and CD64 positive cells)	activate of NK cell promote antibody-dependent cytotoxicity cascade	
		M2-TAMs (CD163, CD206 and ARG1 positive cells)		promote neo-angiogenesis, the secretion of growth factors, the degradation of extracellular matrix (ECM), and the epithelial-mesenchymal transition (EMT)
	TAFs (tumor associated fibroblast )			promote the secretion of IL-6, IL-8 FGF-b, TGF-b, VEGF, CCL2, CCL11, CCL22
	Folliculo-stellate cells	Astrocyte-like FS cells Dendritic cell-like FS cells Epithelial cell-like FS cells		promote the secretion of IL-6, FGF-b, TGF-b, VEGF, leukaemia inhibitory factor (LIF), macrophage inhibitory factor (MIF)
**Immune-check point molecules**	PD-1			neutralize T cells functions
	PD-L1			inhibit CD8+ cytotoxicity T cells
	CTLA-4			
	LAG-3			bind the complexes of antigen and to the MHC-II, leading the T cell suppression
**Cytokines and chemokines**	TGF-α and TGF-β1			promote tumor IL-6 secretion
	IL-10, CCL17, CCL2, IL-1 a, IL-4, TGF-b, INF-g			promote the TAMs polarization to M2-phenotype modulate the tumor infiltration of B cells and CD8+ T lymphocytes.
	IL-6			promote secretion of VEGF-A promote hormone release, tumor cells growth and proliferation
	IL-2			promote GH3 cells proliferation
**Neoangiogenesis**	VEGF/VEGF-R pathway			promote tumor neo-angiogenesis
**Extracellular matrix (ECM)**	MMPs	MMP-1, -2, -9, -14, and -15	Reduce hormone secretion and cell proliferation	Degrade and remodel of the ECM

Somatotropinomas were reported as the subtype of pituitary adenomas/PitNets with the higher number of tumor­infiltrating lymphocytes (TILs) and tumor­associated macrophages (TAMs) [19–21]. Lu et al. described that CD4+ and CD8+ cytotoxic T lymphocytes were more numerous in GH-secreting pituitary adenomas/PitNets than in null cell and corticotroph ones [19]. Similarly, Zhou et al. proved that the total number of all the immune cells was significantly higher in somatotropinomas as compared to not-secreting pituitary adenomas/PitNets [21].

The exact composition and function of the immune landscape in somatotropinomas remains still not fully clarified. To date, several studies were conducted on a heterogeneous cohort for subtypes of pituitary adenomas/PitNets. The data on component of TME in somatotropinomas are summarized in Table 2.

**Table 2:** TME components in somatotropinomas and their actions

**TME components**		**Anti-tumor immune response**	**Immune response escape**
Hormones	GH/IGF-I	Promotion in secretion of IFN-γ and in proliferation neutrophils	Reduction in secretion of TNF-α, IL-6, IL-8, IL-1 β, IL17, IL22
	PRL(acute secretion)	Activation of B cells and neutrophils Promotion in the production of autoantibody Promoting differentiation of dendritic cell in a pro-inflammatory phenotype	-
**Immune cells**	CD8+ cytotoxicity lymphocytes	Reduction of tumor invasive growth Association with good response to SSA therapy	-
	CD68+ macrophages	-	M2-TAMs Positive association with tumor size
	Folliculo-stallate (FS) cells	-	Association to high GH levels, in cases with few widely sparse or scattered cells Promotion in secretion of IL-6
**Immune-check pointmolecules**	Overexpression of PD-L1	-	Inhibition of CD8+ cytotoxicity T cells
	Overexpression of PD-1	-	Association with poor response to SSA therapy
**Cytokines and chemokines**	TGF-α and TGF-β1	-	Promotion of tumor IL-6 secretion
	IL-6	Promotion in the production of PRL	Promotion in secretion of angiogenic factors, such as the VEGF-A Promotion in hormone release, tumor cells growth and proliferation
	IL-2	Promotion in the production of PRL	Promotion of GH3 cells proliferation
	IFN-α	Inhibition of hormone secretion	-
**Extracellular matrix**	MMPs	Reduction of hormone secretion and cell proliferation	-

## Tumor microenvironment (TME)

### Immune cells

The population of immune cells (mainly TAMs and TILs) is the most studied TME component in pituitary adenomas/PitNets. T cells are reported to dominate the microenvironment across all subtypes of pituitary adenomas/PitNets [22]. Recent studies showed that CD68+ macrophages predominate the immune infiltration in pituitary adenomas/PitNets [23].

The immune cells have an heterogeneous behavior in tumor infiltrate. TILs show, in fact, several phenotypes. Cytotoxic CD8+CD45RO+ memory T cells, CD4+ T helper 1 T cells, innate cytotoxic lymphocytes and natural killer cells are generally considered to be anti-tumorigenic and beneficial to the host because they directly interact with tumor antigens and promote a cytotoxic cascade that eventually kills the tumor cells [24]. Cytotoxic TILs also promote the humoral response by an antibody-dependent target cell killing. On the other hand, suppressor (or regulatory) TILs include CD4+CD25+Foxp3+ regulatory T cells, CD4+ T helper 2 T cell and T helper 17 cells [25–27] and are detrimental for the host [24], inhibiting the effector lymphocytes in both cytokine or both cell-contact dependent fashion, and ultimately dampening their beneficial antitumor activities [28, 29].

In a recent multicenter experience that also involved our research unit, the tumor infiltration CD8+ lymphocytes seemed to act as prognostic factors for tumor invasion and therapy outcome. In particular, among a cohort of 64 acromegaly patients, CD8+ lymphocytes were significantly less numerous in tumors with cavernous sinus invasion and in cases resistant to therapy with first-generation somatostatin analogues (SSAs) [30].

The TAMs may polarize into M1-TAMs and M2-TAMs. The M1-TAMs typically express CD80, CD86, MHC II and CD64 and usually inhibit tumors through reactive oxygen species, NK cell activation and antibody-dependent cytotoxicity cascade. The M2-TAMs typically express CD163, CD206 and ARG1 and act promoting the immune response escape, the immune suppression, the neo-angiogenesis, the secretion of growth factors, the degradation of extracellular matrix (ECM), and the epithelial-mesenchymal transition (EMT) [31].

The pro- and anti-cancer roles of TILs and TAMs are not firmly defined and may change in the different tumor stages. Jun and co-authors proposed a time-dependent hypothesis to explain the heterogeneity of tumor infiltrating immune cells [32]. At an initial stage, the tumor cells are recognized and cleared by the immune cells. During the tumor progression, a phase of balance between tumor cells and immune cells may occur. Eventually, the immune response is evaded by the tumor cells, and the immune system is unable to cope with the tumors [32]. As the tumor differentiates, tumor cells may evade the immune system, leading to further cancer spread, infiltration, and even metastasis.

One of the mechanisms that promotes the TME shift toward an immune response escape is hypoxia, that induces several biochemical reactions leading to localized acidification. A high concentration of lactates was found within the anaerobic tumor environment [33]. The lactates have been reported as key signaling molecules of metabolic pathways, immune responses and intracellular communication within the TME [33]. Lactates alter macrophages to acquire properties that enhance tumor growth [33]. Zhang et al. proved in a recent study that the lactate-induced acidification of TME may reshape TAMs into an M2-type phenotype and, indirectly, may promote the activation of the CCL17/CCR4/ mTORC1 axis to enhance tumor invasion [34].

Lu et al. reported that the number of CD68+ cells was positively correlated with tumor size and the high Knosp’s grades [19]. The authors also found that CD68+ cells were more numerous in sparsely granulated somatotropinomas as compared to densely granulated ones [19], that are recognized for their better prognosis and good response to conventional therapies [35].

TME is also composed from tumor-associated fibroblasts (TAFs), cytokines, chemokines, proteolytic enzymes, macromolecules of the extracellular matrix and their receptors, and blood vessels.

The folliculo-stellate (FS) cells are resident non-endocrine cells that comprise 5–10% of the normal adeno-pituitary and are also found in the TME of pituitary adenomas/PitNets [11]. The FS cells are differentiated in three main subtypes: astrocyte-like, dendritic cell-like and epithelial cell-like [11]. In the normal adeno-pituitary, the FS cells regulate the hormone secretion [36], the neuroendocrine response to inflammation and immune stress [11], the microcirculation of ions, nutrients, and waste products [37]. The FS cells are further associated with the production of cytokines and growth factors, such as the IL-6, the follistatin, the basic fibroblast growth factor (FGF-b), the transforming growth factor β (TGF-b), the VEGF, the leukaemia inhibitory factor (LIF) and the macrophage inhibitory factor (MIF) [38]. In one of the biggest studies to date, Voit et al. investigated the FS cells in 286 somatotropinomas showing that 69% of these tumors contained FS cells and that tumors with few widely sparse or scattered FS cells secrete more GH than tumors lacking FS cells [39].

TAFs are targets also for the SSAs. The effects of SSAs on TAFs and other TME components might explain the reason for the higher efficacy of Pasireotide Lar as compared to the efficacy of octreotide, as described in *in vivo* studies on acromegaly patients [40–42]. Instead, *in vitro* experiments showed a similar efficacy of pasireotide and octreotide in inhibiting the growth of pituitary tumor cells [43, 44].

### Immune check-point molecules

The immune checkpoints regulate the immune activity and play an important role in maintaining self-tolerance, controlling the intensity of immune response and in reducing the tissue damage [45].

Immune checkpoints may be expressed by tumor cells and suppress the activation of T cell. The cytotoxic T lymphocyte-associated protein 4 (CTLA-4) and the programmed cell death 1 (PD-1) are the most known pathways in cancers. CTLA-4 limits the CD4+T cell phenotype [46, 47]. PD-1 signaling pathway induces T cell dysfunction, failure and neutralization in tumors [48].

The overexpression of PD-L1 in tumors was a strategy to protect itself from the immune response. Juneja et al. proved that the expression of PD-L1 on tumor cells inhibit CD8+ T cell cytotoxicity, promoting the immune surveillance escape [49]. With this in mind drugs that block immune checkpoints have represented a revolution for the treatment of several types of tumors [50], obtaining promising results in term of overall survival and progression-free time [51].

Preliminary studies were conducted on mice models by Hanna et co-workers, suggesting a novel theoretical rationale for also using immunotherapy in aggressive pituitary adenomas/PitNets. Anti-PD-L1 monoclonal antibody was able to reduce the secretion of ACTH and the growth rate of pituitary adenomas/PitNets, improving the overall survival in murine models [52]. In human treatment Sol et al. reported a patient with ACTH- secreting pituitary carcinoma who obtained clinical remission by a combination therapy with anti-CTLA-4 and anti-PD-1 monoclonal antibodies [53]. Other immune checkpoints (such as LAG3) are also potential targets for immunotherapy in aggressive pituitary adenomas/PitNets. LAG3 binds to the complexes of antigen and to the MHC-II, leading the T cell suppression [20].

The expression of PD-L1 in somatotropinomas remains controversial. Mei et co-authors reported an increased expression of PD-L1 RNA transcripts in 12 somatotropinomas, as compared to those identified in null cell and silent gonadotroph tumors [54]. Wang et al. confirmed the over-expression of PD-L1 though immunohistochemistry in a cohort of 28 somatotropinomas, suggesting that the PD-L1 blockage can be a possible treatment for acromegaly patients [55]. The high expression of PD-1/PD-L1 molecules in somatotropinomas suggests a reduced immune response, including those cases with a high infiltration of CD8+ T cells, justifying an aggressive tumor phenotype [55]. Preliminary data are available for LAG3: in 12 somatotroph tumors, an increased expression of LAG3 was identified with respect of other pituitary adenomas/PitNets [20].

### Cytokines and chemokines system and other soluble factors

Marques et al. proved that cytokines and chemokines may promote the immune cells recruitment [23]. At the same time cancer cells may transform the normal chemokine system, through the production of cytokines that stimulate the neo-angiogenesis and the remodeling of the extra-cellular matrix, promoting the tumor progression [23, 56].

Recent studies have provided that the IFN, the ILs and the tumor necrosis factor TNF play a key role in the differentiation of the pituitary gland and in the oncogenesis of pituitary adenomas/PitNets [57, 58].

The IL-10 family comprises of immunosuppressive cytokines that promote tumor escape from immune surveillance [21, 59], promoting the TAMs polarization to M2-phenotype [31] and modulating the tumor infiltration of B cells and CD8+ T lymphocytes [21].

The chemokines (CCL17 and CCL22), the IL-1a and IL4, the TGF- b and the INF- γ seem to act in the same way. T helper type 1 (Th1) cells secrete TNF- α, IFN-γ, and IL-2; whereas Th2 cells secrete IL-4, IL-5, IL-6, IL-10, and IL-13 [59].

The GH and the IGF-I modulate the secretion of cytokines. IGF-I attenuates the production of monocyte-derived pro-inflammatory cytokines, such as the TNF-α, the IL-6, IL-8 and IL-1β, via the mitogen-activated protein kinase (MAPK). Moreover, the IGF-I promotes the secretion of the IFN-γ and of the IL-17 and IL-22) [60].

Several cytokines affect the release of adeno-pituitary hormones.

In somatotropinomas, the transforming growth factor (TGF)-α and the TGF-β1 stimulate the tumor secretion of IL-6. In parallel, octreotide suppresses the IL-6 secretion [61]. The receptor for IL-6 possesses a binding domain similar to those of the GH, prolactin and erythropoietin [62, 63].

The role of IL-6 in TME of pituitary adenomas/PitNets has not been completely clarified. The IL-6 may contribute to the hormone release, to tumor growth and proliferation and to the production of angiogenic factors, such as the vascular endothelial growth factor-A (VEGF-A) [64–67].

Recently, IL-6 has been shown to stimulate the growth of GH3 rat pituitary tumor cells but to inhibit the growth of normal pituitary cells in rats. The IL-6 reaches the pituitary through systemic blood circulation but it is also intrinsically synthetized and released in the pituitary with a paracrine effect. In the normal adeno- pituitary the major or even exclusive source of IL-6 are the FS cells, under the regulation of the TNF-α. The role of FS cells as a source of IL-6 production is still a matter of debate [60].

The macrophage migration inhibitory factor (MIF) was recently rediscovered as a cytokine and a glucocorticoid-induced immune-modulator, that enhances the production of other inflammatory cytokines as IL-1, IFN and TNF [68]. The secretion of MIF may be induced also by pituitary hormones.

A high expression of CCL2, CXCL10, CX3CL1, with a low number of infiltrating FOXP3 T-cells and a high number of infiltrating CD4+ T-cells was detected in highly vascularized pituitary adenomas/PitNets [56]. The CCL2, which recruits macrophages, was significantly correlated with the micro-vessel area in pituitary tumors [56]. Chemokines and growth factors induce the epithelian-mesenchymal transition (EMT), that is a process whereby tumor cells are reprogrammed to a mesenchymal phenotype, acquiring migratory and invasive characteristics by losing epithelial polarity and adhesion molecules, in particular E-cadherin.

Marques et al. conducted a study on GH3 cells (extracted from 5 cases of human somatotropinomas) and proved that TAFs (tumor associated fibroblast) are able to secrete the CCL2, CCL11, CCL22, VEGF-A, IL-6, IL-8 and FGF-2 [69]. Interestingly the secretion of CCL2 was further increased in GH3 cells that derived from highly vascularized and highly proliferative pituitary adenomas/PitNets [69]. In the same study, the authors showed that the administration of Pasireotide reduced the secretion of IL-6 and of CCL2 [69]. The inhibitory effect of Pasireotide on the secretion of IL-6 may play a role in the clinical effectiveness of this SSA.

Hofland et coauthors moreover showed that IFN-α inhibits hormone secretion and reduces the intracellular hormone concentration in human somatotropinomas [70]. *In vitro* studies proved that also IL-2 may stimulate the proliferation of GH3 cells [71].

The data on anti-pituitary antibodies are few. Lupi et al. detected by immunofluorescence the presence of anti-pituitary antibodies in three out of 68 patients with GH-secreting adenoma (4.4%) [72].

Comparative data on TME in pure somatotropinomas and in mixed somato-lactotropinomas are limited. However, an effect of PRL on TME should be speculated. In fact, PRL bears a structural relationship with members of the cytokine/hemopoietin family (such as IL-2 and IL-17), growth hormones and macrophage colony-stimulating factors [73] . In parallel, the PRL receptors belong to the superfamily of cytokine/hemopoietic receptors and are expressed on the surfaces of the immune cells [74]. PRL activates T cells, B cells, neutrophils, macrophages and stimulates the production of autoantibody. Hyperprolactinemia affects dendritic cell function, switching from an antigen-presenting to a pro-inflammatory phenotype [75, 76] In parallel, same cytokines (IL-1, -2, and -6) stimulate the secretion of PRL [77]. The abnormal local PRL production by immune cells may explain the relationship between PRL and the onset of autoimmune disease [78]. However, data on the effect of long-term hyperprolactinemia are not conclusive. In fact, chronically elevated serum PRL concentration seem not to stimulate the immune system. In fact, the acute elevation of PRL may affect the immune response, instead the persistence of hyperprolactinemia may induce adaptive changes [79].

Among the soluble factors in TME, the interest toward the D3 vitamin is progressively increased. The 1,25(OH)2D3 interacts with the immune system in many different ways, finally enhancing an efficient immune response toward the not-self antigens and enhancing a tolerogenic profile toward self-antigens.

The immune cells express the receptors for D3 vitamin and for the hydroxylase enzymes that induce the hydroxylation of D3 vitamin on the 25 and the 1a sites. The immune cells are able to activate 25(OH)D3 to 1,25(OH)2D3 that acts as a paracrine hormone within the TME. The 1,25(OH)2D3 down-regulates the expression of the costimulatory molecules, such as CD40, CD80, CD68 and MHC-II from the cytomembranes of antigen-presenting cells. The 1,25(OH)2D3 acts directly on T-lymphocytes, regulating the expression of chemokine receptors [80] and inhibiting the production of several cytokines, such as INF-γ, IL-12, IL-17; or stimulating the production of IL-4 [68]. The complex actions of 1,25(OH)2D3 on dendritic cells and T lymphocytes promotes the polarization of T cells from the inflammatory Th1 phenotype to the protective Th2 phenotype, inducing the activations of T regulatory cells [81].

Although *in-vivo* data on the effect of vitamin D deficiency in pituitary adenoma/PitNets are few, in a cohort of 67 female patients with prolactinoma, significantly lower 25-hydroxyvitamin D levels were detected in patients with large tumors [82]. A recent study reported an inverse correlation between the serum levels of 25(OH)D and the concentration of urinary free cortisol in 50 patients with Cushing’s disease [83].

Recently it was suggested that the systemic markers of inflammation may reflect the activation of tumor immune response and may predict the outcome of several types of cancers, including pituitary adenomas/PitNets [84]. The full blood count (FBC), the C-reactive protein (CRP), the albumin and the serum inflammation-based scores have been investigated as markers of systemic inflammation in patients with pituitary adenomas/PitNets [84]. A high neutrophil-to-lymphocyte ratio (NLR), a low prognostic nutrition index (PNI), many leucocytes, neutrophils, monocytes, and few platelets were observed in patients with pituitary adenomas/PitNets as compared to health controls [85]. The secretome of pituitary adenomas/PitNets may remarkably influence the hematopoiesis and the degree of systemic inflammation markers [13].

The GH and the IGF-I increase the neutrophil activation and proliferation via the granulocyte colony-stimulating factor (GCS-F) [86]. Preliminary data are available on acromegaly patients and are mainly focused on the effect of SSA therapy: Szydelko et al. proved a reduction of the white blood cells and of the neutrophil counts after SSA therapy [87].

### The neo-angiogenesis

The tumor neo-angiogenesis is a very complex process that involves numerous molecules and signaling pathways. The pathway of VEGF/VEGF-receptor (VEGFR) is crucial for the neo-angiogenesis and remains the most studied and targeted so far [88, 89]. Meanwhile, M2 macrophages also take part in the neo-angiogenesis in pituitary adenoma/PitNets, together with B cells, CD4+ T-cells and Foxp3+ lymphocytes [56]. Many studies reported that M2-TAMs in pituitary adenoma/PitNets were positively correlated with the micro-vessel density and with the VEGF expression ^31^. Data from Lloyd et al. (on 148 pituitary adenomas/PitNets and 6 pituitary carcinomas) [90] and from Vidal et al. [91] (on 157 pituitary adenomas/PitNets and 7 pituitary carcinomas) supported the theory that benign pituitary adenomas/PitNets are generally less vascularized than the normal pituitary gland and that pituitary carcinomas are more vascularized than pituitary adenomas/PitNets. The results of tumor neo-angiogenesis in pituitary adenomas/PitNets have yielded contradictory and not-conclusive data [92, 93]

Data on neo-angiogenesis in somatotropinomas are scarce. A possible effect of octreotide on the neo-angiogenesis was presumed in somatotropinomas, as it was observed in diabetic retinopathy [18]. Diabetic retinopathy is a micro-vascular disorder due to an aberrant angiogenesis. Neuroprotective substances may prevent the release of VEGF and the subsequent microvascular alterations [94]. In the early phase of diabetic retinopathy, the neuronal damage plays a primary role [18]. *In-vitro* experiments were conducted on retinal explants of mice with diabetic retinopathy [30]. Amato and coworkers showed that treatment with octreotide increased the autophagic flux, through the downregulation of the mTOR pathway, and rescued retinal cells from apoptosis [95]. It may be assumed that the octreotide mechanism of action may also reduce the risk of pathogenic angiogenesis, in pituitary adenomas/PitNets. Preclinical and clinical studies are required to confirm this hypothesis.

### The extracellular matrix (ECM)

The ECM is a network of macromolecules (such as proteins, glycosaminoglycans, proteoglycans, and glycoproteins) in which soluble molecules, such as growth factors and chemokines, are embedded. The ECM takes a central role in the mechanisms of cell proliferation, growth, adhesion, polarization, migration, survival, and apoptosis, both in physiological and in pathological conditions [15].

The endocrine and non-endocrine cells of the adeno-pituitary regulate the ECM composition, that appears consequently to be different in the normal adeno-pituitary and in pituitary adenomas/PitNets [15].

In pituitary adenomas/PitNets, the large majority of studies on ECM focused on the expression of matrix metalloproteinases (MMPs), a family of enzymes that are involved in the degradation and remodeling of the ECM [25]. Different researches have demonstrated that the expression of MMP-1, -2, -9, -14, and -15 was increased both in genomic and in transcriptomic analysis [26, 96]. In GH3 pituitary tumor cell lines, the inhibition of MMPs reduces the hormone secretion and the cell proliferation [97].

## Immunotherapy and targeted therapy

The knowledge of TME may promote the development of new treatments for pituitary adenomas/PitNets, considered refractory to conventional treatments [98].

The research on immune TME has promoted the discovery and the introduction of treatment with immuno-check points inhibitors (ICIs) in clinical practice, revolutionizing the therapy of several malignancies in the last ten years [99].

Considering this ICIs may represent the newest therapeutic option to be studied in aggressive pituitary adenoma/PitNets and in more rare pituitary carcinomas [15].

The rationale behind the use of ICIs is based on preliminary studies that reported the presence of TILs [19] and the expression of PDL1 in pituitary adenomas/PitNets, that are targets of ICIs [54, 55].

*In-vivo* preclinical data have provided promising results of the efficacy of ICIs in murine models of pituitary adenomas/PitNets [52]. In fact, the same clinical trials (NCT04042753, NCT02721732 and NCT02834013) are on-going to provide more evidence on the efficacy and safety of ICIs in patients with aggressive pituitary adenomas/PitNets and carcinomas. In addition, same clinical cases reported the use of ICIs in only corticotroph and lactotroph aggressive pituitary adenomas/PitNets and pituitary carcinoma [100–104].

Despite cases reported in Literature being few, an increased therapeutic efficacy seems to be proved by the combined ICIs (anti-PD1 plus an anti-CTLA-4) treatment regimen [103].

To our knowledge, until now, there are no reports of patients with somatotroph tumors that were treated with ICIs.

Clinical studies on tumor microenvironment (TME) are advocated to rationalize the use of ICIs in aggressive PitNets.

Recently, specific genes related to tumor microenvironment (such as NCAM1, CAM1, CX3CR1. CCL3, CCL4, CCR5, CXCL10, CCR1, CXCL2, PD-L1, STAT3, IRF1, IRF6, IRF8, CTAG2 and TSPYL6) were identified to be overexpressed in PitNets [21, 105–107], suggesting new horizons for immunotherapy.

Target therapy may modulate the TME. Everolimus (an mTOR inhibitor) and bevacizumab (a monoclonal antibody against VEGF) have been the main target therapies that have been reported for the treatment of aggressive pituitary adenomas/PitNEts [15]. The use of target therapies is more experienced in lactotropinomas and corticotropinomas, due to their potentially more aggressive course [4]. The TKIs are a family of drugs that hinder targeted proteins, such as the epidermal growth factor receptor (EGFR), the anaplastic lymphoma kinase (ALK), the breakpoint cluster region–Abelson kinase (BCR–ABL) and the VEGF receptor (VEGFR) [108]. In pituitary adenomas/PitNets, a potential therapeutic role of the EGFR pathway inhibitors has been supported by the results of “*in vitro*” and “*in vivo*” preclinical studies and clinical models [109]. The PI3K–AKT–mTOR pathway seem to be upregulated in pituitary adenomas/PitNets [110–112], with an *in vitro* and *in vivo* anti-tumor effects [113–116].

Clinical trials and case-series on target therapies in somatotropinomas are very limited. To our knowledge, therapy with VEGF inhibitors (bevacizumab and apatinib) was described in only two patients affected by acromegaly [117] and gigantism [118]. *In-vitro* studies on cell cultures of humans and mice somatotropinomas provided encouraging results on the use of mTOR inhibitors, with the reduction of GH secretion and the induction of cell death [113, 117, 119].

## Conclusions

**Fig. 1:**
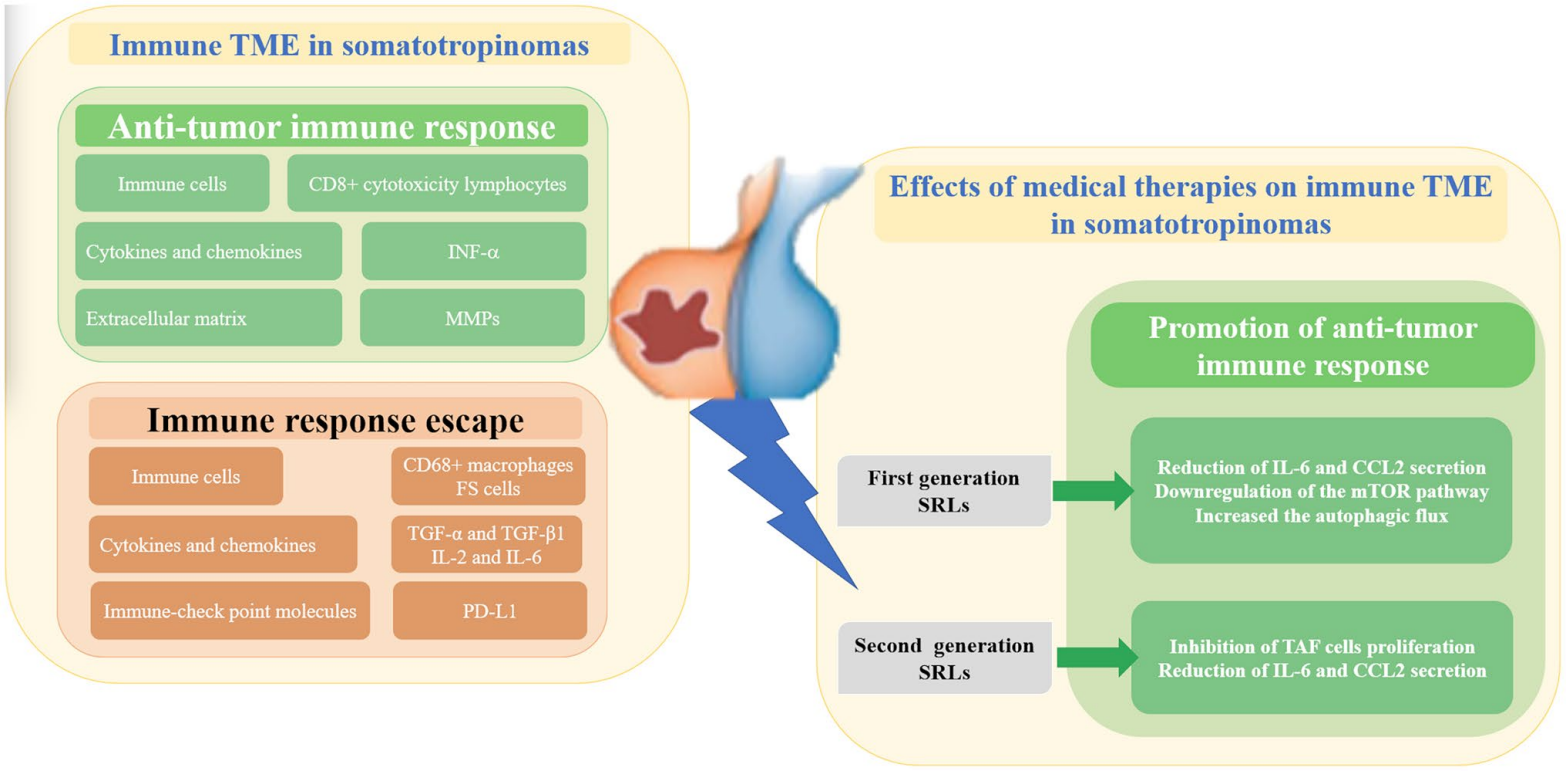
Representative picture of the TME components in somatotropinomas and of the effect of treatment with somatostatin analogues

The mechanism of pituitary adenomas/PitNets, their occurrence and development is still unclear, which may be a result of multiple factors such as epigenetics, genes and TME. The immune TME may represent a new scenario for understanding the heterogeneity of tumor behavior and for predicting the outcome of treatments. The data on the TME in somatotropinomas and on effects of the somatostatin analogues are very limited, as shown in Fig. 1. Future studies are advocated to characterize the TME in the different groups of pituitary adenomas/PitNets and particularly in somatotropinomas, prior to understanding its exact role and action. New immune biomarkers may be integrated into those that are actually available, such as the Ki-67, the somatostatin receptor and the cytokeratin pattern, for a personalized therapy, based on the patient’s profile [35, 120] and may promote the use of personalized and target therapy in aggressive and multi-drug resistant somatotropinomas, such as VEGF and m-TOR inhibitors and immune-therapy with monoclonal antibodies against immune-check points.

## Data Availability

Data sharing not applicable to this article as no datasets were generated or analysed during the current study.

## Abbreviations

CD: cluster of differentiation
CTLA-4: Cytotoxic T-Lymphocyte Antigen 4
ESE: Endocrine society of Endocrinology
FS cells: folliculo-stellate cells
GH: growth hormone
GPS: Glasgow Prognostic Score
HLA: Human Leukocyte Antigens
HPF: high powered field
ICIs: immune-check point inhibitors
IGF-I: insulin like growth factor-I
IHC: immunohistochemistry
IL: interleukin
INF: interferon
LAG3: Lymphocyte Activating 3
LAMP2: Lysosomal-Associated Membrane Protein 2
LDHA: Lactate Dehydrogenase A
LMR: Lymphocyte-to-Monocyte Ratio
MAPK: mitogen-activated protein kinase
MIF: macrophage inhibitory factor
NET: neuroendocrine tumors
NLR: Neutrophil-to-Lymphocyte Ratio
NPS: Neutrophil-Platelet Score
OGTT: Oral glucose tolerance test
PA: Pituitary adenoma
PCR: Polymerase chain reaction
PD: Programmed cell death
PD-L1: Programmed cell death ligand 1
PLR: Platelet-to-Lymphocyte Ratio
PNI: Prognostic Nutrition Index
PRL: prolactin
SSAs: somatostatin analogues
SII: Systemic Immune-Inflammation Index
TAMs: tumor­associated macrophages
TGF: transforming growth factor
TILs: tumor­infiltrating lymphocytes
TIM3: T-cell immunoglobulin domain and mucin domain 3
TIME: tumor immune microenvironment
TME: tumor microenvironment
TNF: tumor necrosis factor
VEGF-A: vascular endothelial growth factor-A
